# A classification of the use of research indicators

**DOI:** 10.1007/s11192-016-1904-7

**Published:** 2016-03-23

**Authors:** Joost Kosten

**Affiliations:** Centre for Science and Technology Studies, Leiden University, P.O. Box 905, 2300 AX Leiden, The Netherlands

**Keywords:** Research assessment, Research and innovation policy, Classification, Literature study

## Abstract

Research performance indicators are broadly used, for a range of purposes. The scientific literature on research indicators has a strong methodological focus. There is no comprehensive overview or classification of the use of such indicators. In this paper we give such a classification of research indicator use. Using the journal *Scientometrics* as a starting point we scrutinized recent journal literature on scientometrics, bibliometrics, research policy, research evaluation, and higher education in order to spot paragraphs or sections that mention indicator use. This led to a classification of research indicator use with 21 categories which can be grouped into five main categories.

**Electronic supplementary material:**

The online version of this article (doi:10.1007/s11192-016-1904-7) contains supplementary material, which is available to authorized users.

## Introduction

The use of research performance indicators is often discussed in the literature on science and technology policy. Such discussions focus on, amongst others, the optimal measurement of research performance, research impact and research quality and on the possibly detrimental consequences of improper use of research indicators (e.g. Hicks et al. 2015). Many researchers seem to take for granted that there is an ever increasing level of use of research performance indicators, but actually there is no systematic analysis of the level of use. A first step towards such a systematic study would be a qualitative classification of the *types* of use. The literature provides a few studies on some types of use, but usually these merely aim to propose an alternative indicator or concentrate on a particular indicator or evaluation system which includes research performance indicators.

The purpose of the present paper is to develop a comprehensive classification of the types of use of research indicators which can be used in quantitative studies. The classification is derived from the information on research indicator use given in the journal literature in the field of scientometrics/bibliometrics. We illustrate the different categories in the classification by means of examples of the mentions of use from which the classification has been derived. In this way, we will also give an overview of recent studies which mention the use of research performance indicators.

In this paper, we use the term indicator in the sense of a quantitative characterization of research performance. This does not necessarily mean that an indicator should be based on measurement, it can also result from qualitative assessments. Moreover, we focus on research *performance* indicators, or research indicators for briefness. Therefore, we do not include the use of input indicators such as the number of staff nor the number of PhD students. In contrast, indicators such as third-party funding are included if they are used as a proxy for quality.

We collected relevant studies containing references to the use of research indicators primarily from *Scientometrics*. This journal has an established position in the field and pays much attention to research indicators. In order to create a classification which is based on the most up-to-date information, we focussed on the most recent period (2003–2013). Recent volumes of other journals have been used to verify whether the information on indicator use they provide also fits the initial classification. Some of these journals were in adjacent fields such as research and science policy, and research evaluation.

Though the present paper is the first to focus on indicator use, there have been comparative studies of other subjects that provided information on indicator use as a collateral: studies of funding systems, organization and management and, in one case, of rankings. These studies with collateral comparative information on indicator use are reviewed in the Online Resource section 2.

This paper is structured as follows. In the “Methods and data collection” section we set out our methodology. The “Classification” section is devoted to the classification itself. The “Concluding remarks” section draws some conclusions about the available literature on the use of research performance indicators and provides some reflections on the usefulness of the classification and future work.

## Methods and data collection

Naturally, the development of a classification of the use of research indicators requires as a ‘data source’ some kind of inventory of the various types of use to which indicators have been put. The literature contains no recent comprehensive overview of indicator use. Thus far, the recent literature only provides overviews of some areas of use (see Only Resource section 1). Therefore, we had to develop our own data source. In principle, this could be done in various ways. Since use can take many different forms, one approach would be to consider papers of organizations that use the indicators: universities and other research organizations, funding organizations, governments, international organizations, and so on. In fact, to obtain a quantitative picture of indicator use, this might be the only valid approach. However, for the purpose of the design of a classification, this approach has three problems. Firstly, the selection of the ‘users’ would be rather arbitrary. Secondly, the selection of the ‘papers’ of the use would be subjective and might easily cause biases. And finally, the approach would probably be much more laborious than the development of a classification requires, since the question guiding this research is not how often a type of use occurs but which types of use can be distinguished, which precedes the other question. Therefore, another approach has been chosen, namely to use a well-defined scientific body of literature as source. The topic and size of this body of literature should be such that it covers the most important topics regarding the use of research indicators. We are only interested in the *types* of research indicator use and not in how often those types of indicator use are mentioned in the literature or applied in practice. Therefore it does not matter if some papers referring to research indicator use are missed by the searching methods we apply as long as all *types* of indicator use are mentioned at least once.

### Scientometrics

Given the focus on (bibliometric and scientometric) research indicators, the journal *Scientometrics* was selected as a primary source.^1^ Given *Scientometrics*’ focus on “quantitative features and characteristics of science and scientific research” and its role as “indispensable [journal] to research workers and research administrators” and “provide[r of] valuable assistance to librarians and documentalists in central scientific agencies, ministries, research institutes and laboratories” (Scientometrics 2014). Including descriptions of practices which are clearly obsolete nowadays would not make sense. Therefore, only papers published in the volumes of *Scientometrics* during the period January 2003 till March 2013 have been analyzed (vol. 56(1)–94(3)).

From this body of papers, the contributions containing information on indicator use were selected as follows. Identification of relevant papers started by browsing 75 *Scientometrics* issues published from 2007 until 2013 (vol. 70 until vol. 94) manually. Every article of which the title or the abstract provided an indication that it could contain a description of the use of research indicators was scrutinized in order to ascertain if it actually contained such a description. Once information about the application of research indicators was found, the paper was used as a source of information for the classification. To be included in our collection, descriptions should be clear and specific enough to know where and in which context research indicators are used. The context in which such a description is given did not matter. Therefore, our selection includes papers by authors whose work does not focus on research indicator use and who might themselves use other sources to describe the use of indicators. In this way, the 26 articles selected for further analysis form a subset of all papers in *Scientometrics* in vol. 70–94.

After this first group of articles had been identified, we enlarged the set of papers used as source for the classification by means of a keyword search. This approach is less time consuming than a ‘manual’ search while it would not necessarily yield less relevant results, provided the keywords have sufficient specificity. Since a combination of the keywords ‘research’ and ‘evaluation’ gives results about research evaluation, but any kind of research on evaluation, such as the results of research on the evaluation of certain types of medication more specific results are needed. Therefore, the selection of keywords that are both sufficiently inclusive and sufficiently discriminatory is of great importance if we want to expand this searching method to a broader set of journals. Eventually, from the initial set of 177 papers resulting from out searching method, we obtained twelve papers by reading them as in the first set of papers. This is less papers per volume than the result of the manual search. More details on our keywords based search method can be found in Online Resource section 2.

### Contributions in other journals

*Scientometrics* in itself might have certain limitations as a source for descriptions of the use of research indicators.^2^ This medium contains mostly papers on methods of bibliometric or scientometric measurement without reference to the actual use of research indicators. Since the use of research indicators may be important for any researcher or scientist, this topic might be covered by any journal. Still, we only had a limited amount of resources available. Therefore, the preliminary results were verified with contributions to eight other journals with a similar or a slightly different focus compared with *Scientometrics*: *Higher Education, JASIST, Journal of Informetrics, Minerva, Research Evaluation*, *Research Policy*, *Science and Public Policy,* and *Social Studies of Science*. Moreover, we analyzed the *PLOS ONE* sections ‘Science policy’, ‘Research assessment’ and ‘Library science’. Papers were selected in the same way as the *Scientometrics* papers. This process resulted in 59 papers with relevant information for the classification. These contributions were used to verify the preliminary classification and to enrich it with additional categories and examples. Online resource section 2 contains an overview of the results per journal; it shows there are considerable differences per journal.

## Classification

This section presents the result of our study: a classification of all types of research indicator use. The classification (Table 1) has two levels. Five main categories of use are identified: general science policy, funding allocation, management and organization, content decisions, and consumer information. Within these categories 21 different types of activities are identified in which research indicators are often used. Table 1 contains both the classification and the description of the different types of use. In the next section we give examples from the literature with more specific information and references to current practices.

**Table 1:** Classification

	(Main) activities	Description
**A**	**General science policy**	**Use of research indicators for information and decision-making on, and for evaluation of, science and research in the long run on an abstract level**.
1	General policy information	Use of research indicators to inform policy makers about the current state of affairs of science and research.
2	Policy formulation	Use of research indicators as a source of information to support decision making, policy development, and the setting of policy goals.
3	Policy evaluation	Use of research indicators to evaluate policies or programs. The results of evaluations can in themselves feed back into policy formulation.
4	Inducement	Use of research indicators to create performance incentives that are not purely or primarily intended to have another function such as solving a budget allocation problem.
**B**	**Funding allocation**	**Use of research indicators for funding allocation. The link between funding and research indicators can take a number of different forms.**
	Block funding	Block funding gives recipient institutions discretion to spend funding according to their own views. We include block funding with some general constraints on the purpose for which it is used (constrained block funding). Two types of block funding are distinguished.
5	- Formula-based block funding	Use of research indicators as a variable in a funding formula next to other variables such as faculty and number of graduates.
6	- Non-formula block funding	Use of research indicators without formulae, but, e.g., in negotiating contracts on which funding is based.
7	Additional funding, financial bonus or penalty	Earning of extra funding by institutions or individuals based on their research performance. A financial bonus or additional funding comes on top of basic funding and is not granted to everyone but only to those eligible. Financial bonuses or penalties can also result from contractual agreements which contain indicators as targets.
8	Program and project funding	Use of research indicators to decide about the funding of research programs or projects on the basis of project proposals. In some systems, indicators play an immediate role in the decision-making, while in other decision-making processes indicators are used to inform peer reviewers or decision makers.
9	Internal funding	Use of research indicators for funding allocation within institutions. Internal funding can reflect external funding mechanisms such as formulae or contracts in which indicators can play a role.
**C**	**Organization and management**	**Indicators are used for a diversity of management and organization activities**.
10	Strategy	Use of research indicators by institutions in the formulation of their strategy, to decide upon such a strategy and to set aims to pursue.
11	Contract-based governance and steering (management by objectives)	Use of research indicators in contracts between ministries and institutions or between institutions and departments to agree upon targets to be met. This concerns cases where no funding is involved.
12	Accountability	Use of research indicators by researchers, research groups, and research institutions to inform on their research activities to their higher management or to society at large.
13	Human resources management	Use of research indicators for the selection, hiring, promotion, and dismissal of personnel.
14	Quality management and quality assessment	Quality assurance or quality improvement cannot only be achieved by funding or inducements, but also by means of quality assessment. Committees assessing quality might use research indicators as information about the research performance.
15	Reputation management	Use of research indicators to advertise strengths of a research institution or individual researchers.
16	Selection of partners and members	Use of research indicators to inform institutions about the research performance of possible partners or candidates which apply for membership of professional associations.
**D**	**Content management and decisions**	**Research indicators can play a role in issues which are immediately linked to the contents of research**.
17	Publication channel selection	Use of indicators such as the journal impact factor by authors to decide in which medium they will try to publish their work.
18	Research profile management	Use of research indicators by research institutions to manage their research profile and evaluate their strengths and weaknesses.
19	Journal and database management	Use of research indicators to manage or support journals and bibliographic databases.
20	Library collection management	Use of research indicators by librarians to inform themselves about publications which should be adopted by their institutions.
E	Consumer information	Research indicators such as rankings are proxies for quality of institutes and serve different groups of consumers as important sources of information.
21	Consumer information (not elsewhere specified)	Use of research indicators as source of information by different types of consumers, e.g., students and their parents about the performance of higher education institutions, or researchers looking for another employer.

### Examples

Table 2 contains a number of important examples to illustrate the various categories. Sources of these examples are both the dataset collected for this analysis and the studies mentioned in the first section of the Online Resource. Forms of use can be assigned to more than one category. A complete overview of all examples which led to the classification can be found in the Online Resource section 3.

**Table 2:** Examples

	Main activities	Examples
A	General science policy	The French president Sarkozy used ranking positions to formulate the goal that two French institutions should be in the top 20 and ten should be in the top 100 by 2012.
B	Funding allocation	In many countries across the world, indicators are used in formulae to allocate block funding. Examples can be found in both the Anglo-Saxon world (e.g., the UK, Australia, New Zealand, South Africa) and continental European countries (e.g., Italy, Flanders, Norway, Denmark). Individual bonuses are rather common. One of the most extreme cases can be found in China, where some universities provide individual bonuses that vary with the impact factor of the journal in which a publication has appeared.
C	Organization and management	Human Resources are (partly) managed by means of indicators. This is the case in the UK, where quality of research output by individual staff members could heavily influence the outcome of the Research Assessment Exercise (RAE). Therefore, job candidates were sometimes asked to provide bibliometric indicators while institutions actively headhunted productive researchers and made retirement of unproductive researchers attractive. In many countries, indicators play a role in scientific career opportunities. For instance, applications for associate professorships in Turkey are only open to researchers with at least one publication in a Web of Science indexed journal.
D	Content management and decisions	Research indicators can serve researchers to help them make decisions on the journal in which they should publish a contribution. The UK-based Association of Business Schools developed a journal ranking with such a purpose in mind.
E	Consumer information	Research evaluation results from the UK RAE and the Turkish ranking of universities are publicly made available to help prospective students and their parents choose an institution for further education.

## Concluding remarks

We analysed the mentioning of use of research indicators in recent issues of a number of international journals on bibliometrics, scientometrics, science policy, research management, and research evaluation. The use of research indicators is not a prominent topic in this scientific literature and often appears as a ‘collateral’ when the theoretical characteristics of research indicators are discussed. Much research focuses on the development and properties of the indicators while disregarding their actual use. By and large, this is true for all journals considered.

Research indicators are used for many different purposes, ranging from high level nationwide policies to micro considerations regarding the choice of a publication outlet. In our classification we have identified five main types of use: General science policy, Funding allocation, Organization and management, Content management and decisions, and Consumer information. Within these main categories, a diversity of specific types of use has been classified and illustrated with recent examples.

There are still a number of questions which deserve further attention. To what extent is the use of research indicators widespread? And if research indicators are used, what is their influence? The former question can be answered by means of quantitative methods while the latter question can be addressed by means of both quantitative as well as qualitative methods. In both cases, our classification is a convenient point of departure. In a way, Hazelkorn (2007) has addressed part of these questions in her survey of the effects of league tables. As indicators are often used for funding in ways that differ quite substantially between countries, further research could prioritize the effects of this type of use.

What are the implications of our findings on indicator use for the grand challenges in data integration? The overview of the current uses of research indicators presented in this paper clearly reveals the relevance of data integration and interoperability: there is such a diversity of uses that no single indicator can serve all of them, nor is it likely that any indicator is useful for just one of these purposes. This means that most purposes can only be served by a combination of indicators and that, at the same time, many indicators will serve several purposes but together with different combinations of other indicators. This, in turn, means that it is highly desirable that data from different sources and different indicators can be related to each other, and share common definitions and classifications so that they can be used in combination. To give one example: anyone who has ever worked with data on universities from different sources knows that it is highly desirable to develop a standard delineation of universities that is used in all sources, so that bibliometric data from various sources as well as altmetric data and financial data can be more easily related to each other.

An obvious way to move forward is to analyze which data sources and indicators are used for which purposes. This means that the data sources and indicators are classified according to our classification of types of use. This will show where comparability or ‘relatability’ is most needed and what type of action has uttermost importance. Thus, if it turns out that a certain set of indicators is often used for the purpose of the allocation of funding to universities, then it makes sense to prioritize the work on a common delineation of universities in this set of indicators and the underlying data sources. Consequently, the grand challenges posed to the current infrastructure can be addressed more easily and even be guided by using our classification of types of use.

## Electronic supplementary material

Below is the link to the electronic supplementary material.

Supplementary material 1 (DOCX 72 kb)

## Footnotes

1 The relevance of *Scientometrics* as selected source of articles can be deduced from the results if one looks up a combination of “research evaluation” or “research assessment” and “indicator” (with a wildcard): a total of 226 results are found, with the largest number of papers in *Scientometrics* (52) and *Research Evaluation* (26). Applied search string in the Web of Science (online): ts = ("research evaluation" or "research assessment") and ts = (indicator*).

2 Although an alternative source of papers might not be obvious, see note 1.
