# Supplementary material for: A classification of the use of research indicators
Source: Scientometrics. 2016 Mar 23;108:457–64. doi: 10.1007/s11192-016-1904-7 (PMC4909787; doi:10.1007/s11192-016-1904-7)
Supplement: Supplementary file 1 — Supplementary material 1 (DOCX 72 kb) [file 11192_2016_1904_MOESM1_ESM.docx]

**A classification of the use of research indicators**

**Journal:** Scientometrics

**Author:** Joost Kosten

**Affiliation:**

Center for Science and Technology Studies, Leiden University

P.O. Box 905

2300 AX Leiden

The Netherlands

**E-mail:** kostenmjf@cwts.leidenuniv.nl

**Phone:** +31 71 527 6104

This supplementary material contains three sections. In the first section, an overview of previous contributions on the use of research indicators is given. In the second section, the keyword based search method as applied for the creation of the classification is presented. Finally, based on the scientific literature the third section contains an overview of examples of research indicator use per category of the classification.

**1. Overview of other contributions**

An overview of contributions on the use of research indicators published from 2000 on and which mention at least two countries or institutions when it comes to research indicator use. Two main topics have been identified: the role of indicators in funding and in management and organization. Moreover, this overview contains a brief description of a study by Hazelkorn (2007) which is entirely devoted to rankings and how they are appreciated and used. The types of use in this overview are discussed in terms of the classification presented in the main paper.

Funding

A number of contributions focus mainly on the use of research indicators in funding allocation. This comes as no surprise. Since funding allocation often takes place within a *national* system, there is a large amount of documentation and great importance is attached to funding in research policy.^[[1]](#footnote-1)^ Within funding systems, there usually is “a mix of funding mechanisms” (Jongbloed, 2011, pp. 179–80). Many of these mechanisms will be mentioned below. We discuss the literature in terms of the classification we developed. Therefore, readers unfamiliar with the relevant technical terms are referred to the section ‘Classification’ for an elaboration.

According to Hansen (2010) two countries have a history of more than two decades with either the peer review based or the metrics based evaluation system. One is the UK with its Research Assessment Exercise (RAE). In the past, Australia used an output measurement based approach (e.g. publications, external funding), while it currently uses ratings based on informed peer review. In many countries, Hansen says, variations on these models have been implemented. A third type of funding system identified by Hansen is funding based on the measurement of citations (measuring effect instead of output) which is used in Flanders. The Flemish model includes publication counts as well. Hansen’s overview contains references to the use of indicators for formula-based block funding (FBBF) (UK, Australia, Belgium (Flanders), Norway, Italy, New Zealand), additional funding (Denmark), and financial bonuses (Spain) (Hansen, 2010, pp. 66–74).

Box (2010) points out that indicators used in PRFSs can have additional functions next to their role in funding. Linking funding to performance, such systems are oriented towards, e.g., improving research quality and fostering research excellence, accountability, and stimulating the decentralization of decision making on science from “politicians and officials” to the research community. Note that these intended effects or objectives of the *funding system* should not be confused with the types of use of research indicators. In these systems, the indicators are used for funding, not directly for the aims of the funding system (Box, 2010, pp. 91–2; 121). Box mentions indicator use for project funding (SL, p. 92), FBBF (Australia, Austria, Belgium, Finland, Germany (partially), New Zealand, Norway, Poland, Sweden, UK, pp. 94-101), additional funding (Denmark), internal funding within institutions (Austria). Box emphasizes that research indicators are often made public. Thus, apparently, indicators are used for accountability to a broader public with respect to performance and funding allocation (pp. 102-3). Moreover, in Denmark indicators (journal impact factors) are used for content-related purposes, namely the ranking of journals. In this way, indicators are used for the evaluation of the content of scientific contributions. Box does not clearly point out why this is done, but probably this is a way to distinguish top journals from average journals for the Danish Bibliometric Research Indicator. For this indicator, more weight is attached to publications in higher rated journals (p. 114).

Jongbloed and Vossensteyn (2001) analyze performance-based funding in higher education in a number of countries. Performance has a limited importance for the funding of higher education (both research and teaching) in the countries they investigated (2001, p. 135). As far as they mention the use of research indicators, the focus is on FBBF (Australia, UK). They mention the use of research performance indicators for accountability and providing information to other stakeholders such as consumer information for students but do not give clear examples (Jongbloed and Vossensteyn, 2001, p. 141).

Geuna and Martin (2003) compare twelve mostly European countries and concentrate on research funding. Research indicators have been used for FBBF (UK, Australia, Slovakia, Poland, Hong Kong), departmental non-formula block funding (Poland), strategy development (The Netherlands), quality assessment (The Netherlands), additional funding (parts of Germany), contract-based governance/steering to meet planned targets (performance agreements) and to increase international collaboration (Finland) and as general policy information (Hungary).

Hicks (2009), (2010), and (2012) also concentrates on the use of research performance indicators for funding. A limitation to performance-based research funding systems (PRFS) is that the heterogeneity regarding where, how, and why research is evaluated “can confound attempts to produce a coherent review” (Hicks, 2012, p. 252). Nevertheless, Hicks mentions research indicator use for FBBF (UK, Australia, New Zealand, Italy, Slovakia, Norway, Sweden, Belgium, Denmark), departmental non-formula block funding (Poland) (Hicks, 2012, p. 259), and bonus funding (Spain) (Hicks, 2010, p. 35). Moreover, sometimes results of PRFSs are publicly available and together with other indicators such as ranking results are used for internal funding (e.g. UK), human resource (Spain), reputation management (Australia), policy formulation (France) (Hicks, 2012, pp. 256–58), and pure inducements (US) (Hicks, 2012, p. 260).

Auranen and Nieminen (2010) analyse if competitive funding and publication activity are correlated. The authors point out that indicators are used for different types of funding. Indicators used for FBBF are the number of publications (Australia), the number and quality of publications (Norway), the number of centers of excellence based at a university (Finland), and the amount of (external) research income (UK, Australia, Finland, Netherlands, Norway, Denmark), and scores from peer review (UK) (Auranen and Nieminen, 2010, pp. 826–7).

Coryn et al. (2007) classifies different funding systems. This comparative study includes many different funding systems, but which “measures of performance” are used and how they are used is not clarified for most examples. An example for which they clearly indicate the use of research indicators is the FBBF mechanism of the now obsolete Australian Relative Funding Model (number of publications) (2007, pp. 441–2).

A study by Laudel (2005) emphasizes the wide-spread use of external funding as indicator for the allocation of research funding. In Australia, external funding is used for FBBF among universities and internal distribution of funding. In the UK, external funding is used for quality assessment in the RAE. In the United States, external funding such as the number of grants won is used for human resource management such as tenure track decisions.

Molas-Gallart (2012) compares the UK and Spain. In the UK, research indicators resulting from the RAE are used for FBBF. In Spain, publication indicators are used for the evaluation of researchers who wish to be granted a *sexenio*, which is an individual-level bonus funding scheme. Having been awarded a *sexenio* is a human resource management instrument in itself to select researchers for promotion and participation in committees. Moreover, sometimes, performance evaluations are used for the development of strategies using “batteries of indicators” (Molas-Gallart, 2012, pp. 591–2).

Performance indicators are used by institutions for internal funding allocation (Liefner, 2003). An example is Bristol University (UK) which distributes funding according to project proposals or high quality research as identified by the Research Assessment Exercise (RAE). Another example is the University of Twente which used, amongst others, the number of employees funded through external funding for its internal-funding model. However, this model has now been superseded by a new one, described by Jongbloed (2011, pp. 183–188). Performance-based funding has been replaced by contract funding. Contracts between the university board and institutional managers contain targets which are formulated as performance indicators to be achieved in a five-year period. In this way, future performance becomes more important. Maastricht University uses similar performance agreements, but these are not used for funding but only for strategy formulation and quality management. In case of Delft University of Technology, performance indicators were used for bonus funding. Bonuses were paid for third-party funding and publication output.

Organization and management

Orr (2004) compares research evaluation in the United Kingdom, The Netherlands, Lower Saxony (Germany), and Ireland. He points out that the results from the RAE are also used for reputation management (“promotional purposes”) and “as a qualitative indicator when competing for third-party funds” (2004, p. 350). In The Netherlands, publication related indicators can be part of evaluation results and are thus used for quality assessment. Individual institutions use such information for internal funding allocation and human resources management (2004, p. 351). In Lower Saxony, research indicators such as third party funding play a role in the funding model (2004, pp. 351–2), which is probably an example of FBBF. Orr does not provide information on the use of research indicators in Ireland.

Simon and Knie (2013) compare the UK RAE, the Dutch Standard Evaluation Protocol (SEP), and the evaluation of the German Leibniz institutes. The authors point out that the German Leibniz institutes report a self-assessment which includes research indicators on publications, conferences and third-party funding. Those results are used for frequently occurring institutional evaluation (2013, pp. 409–10). Evaluations in which research indicators can play a role are used for accountability, funding decisions, and for strategic and organizational recommendations. This is, however, rather indirect use of research indicators.

Ab Iorwerth’s (2005) working paper is a policy advice for Canadian policy makers. He gives a number of examples of research indicator use. Agreements between university and the Ministry of Research in Denmark focus on success criteria and measurement of outputs, which is an example of contract-based governance. With respect to the Netherlands and Switzerland, Ab Iorwerth points out that bibliometric indicators have been used in evaluation of institutes for quality assessment only (Ab Iorwerth, 2005, pp. 46–7).

Butler (2010) focuses on the *effects* of the implementation of performance based funding. In well-known earlier contributions, she already showed convincingly that performance based funding systems can influence publication behavior and thus can have serious effects on performance (Butler, 2003; Butler, 2004). Types of research indicator use she mentions are the use for formula-based block-funding (Australia) (2010, pp. 138–9), the use for strategic decisions such as the closure of departments or institutes after low performance in the UK RAE or in France after the AERES exercise (2010, p. 150). AERES (*Agence d'évaluation de la recherche et de l'enseignement supérieur*) is a French agency which coordinates regular evaluations of research and higher education institutions. Evaluations take place the year prior to the contractual negotiations between the institutions and the French Ministry for Research and Higher Education to provide both parties with common knowledge (AERES, n.d.). Evaluations of research units include grading aspects of (research) performance (Kenna and Berche, 2011, 529; AERES, n.d.).

Survey on the use of rankings

Hazelkorn (2007) employed an alternative approach. In order to examine the impact of league tables and ranking systems (hereafter: LTRS or rankings) on institutional and academic behavior, she undertook a survey among members of the OECD Programme on Instititutional Management in Higher Education (IMHE) and the International Association of Universities (IAU). Those invited encouraged others to participate as well. In the end, her survey included respondents from 202 institutions in different countries. The survey concentrated on the influence of LTRS on institutional decision making, key stakeholders, and higher education.

Respondents believe that the provision of comparative information is the most important purpose of LTRS. Moderately important perceived purposes are to “designate quality”, the measurement of performance, and the promotion of competition. Funding purposes are seen as the least important. Asked about the targeted audience and the targeted users, there are considerable differences. Students are believed to be by far the most important audience of rankings, followed by public opinion. These groups are not believed to be equally important as users: governments, parents, and industry are seen as more important users than audience (2007, p. 97)

Almost half of the respondents use their university ranking position for reputation management. Moreover, rankings are used for institutional management and organization such as strategic or organizational decisions, quality assessment, and decisions on collaboration such as partnerships (staff exchanges, (inter)national collaboration, academic programmes), on human resources management, or on support by other institutions for membership of academic or professional organizations. Many respondents consider rank positions prior to discussing future collaboration with other institutions and even more believe rankings influence the willingness of others to collaborate with them (pp. 97–101).

Hazelkorn did not just ask the respondents how they use rankings, but also examined the perceptions of the respondents on the *impact* of rankings. Many respondents think that funding is not an important purpose of rankings, but many do believe that LTRS have an impact on diverse forms funding as well as on the classification and accreditation of institutions in higher education policy. Asked about the impact of LTRS, the respondents indicated that they provide comparative information, emphasize research strengths, and assist HEIs in strategic planning. Favouring established universities, and openness to distortion and inaccuracies are other properties deemed equally important. Moreover, respondents believe that LTRS establish a hierarchy of HEIs. They mostly do not believe that rankings provide a full overview of an HEI, encourage fair competition, enable HEIs to identify true peers, nor promote institutional diversity. The group of respondents is more divided about the impact of LTRS on performance and quality assessment, accountability, and reputation. Thus, in some respects, LTRS are believed to be helpful organization and management tools, but are equally believed to have considerable limitations (2007, pp. 103–4). Moreover, one of the conclusions Hazelkorn draws is that rankings are nowadays used beyond the original context of “providing comparative information to key audiences, *e.g.* students, public opinion and parents” (2007, p. 107).

**2. Keyword based search method for *Scientometrics***

In order to find additional *Scientometrics* papers which could contain descriptions of the use of research indicators a search string was compiled. The keywords were defined on the basis of the abstracts of already identified papers that were found by browsing the *Scientometrics* issues published from January 2007 until March 2013 (26 papers). A frequency list of the words was made. Numbers were removed, as were comma’s and other forms of interpunction and the possessive ‘s. Given the fact that they are usually part of common terms, hyphens and the ampersand were not removed. Next, general words such as articles, prepositions, pronouns, general adjectives, modal and auxiliary verbs and pro-forms were deleted.

From the remaining list of keywords, terms were selected that distinguished papers related to the use of research indicators from other papers. Within the selected group of terms, three levels of importance were distinguished in order to search as focused as possible and to avoid an overload of irrelevant papers. First order keywords consist of keywords which indicate the sectors in which research indicators are used: research, higher education or science. Keywords of the second order indicate the aspect of research, higher education or science to which articles in the 2007 – 2013 volumes refer: (performance) evaluation or assessment, policy and funding. Finally, the group of third order keywords contains all remaining keywords which either appeared at least 14 times or appeared at least six times *and* were potentially related to the use of research indicators.

The search strings were created in the following way. Every keyword in the first column was combined with two other keywords: either two from the second column ($3\times\binom{5}{2}=3\times10=30$ combinations) or one from the second and one from the third ($3\times5\times24=360$ combinations).

|  | **First order: sector** | **Second order: type of use** | **Third order: other keywords** |
| --- | --- | --- | --- |
| 1 | Research* (83) | Evaluat* (41) | Indicat* (32) |
| 2 | Scien* (52) | Polic* (15) | Publication* (37) |
| 3 | Higher education (7) | Perform* (20) | Universit* (23) |
| 4 |  | Fund* (19) | Number* (20) |
| 5 |  | Assess* (7) | System* (22) |
| 6 |  |  | Bibliometric* (16) |
| 7 |  |  | Journal* (21) |
| 8 |  |  | **Paper* (21)** |
| 9 |  |  | *Review* (18)* |
| 10 |  |  | *Output* (13)* |
| 11 |  |  | *Rank* (24)* |
| 12 |  |  | Result* (14) |
| 13 |  |  | **Stud* (17)** |
| 14 |  |  | *Cit* (19)* |
| 15 |  |  | Impact (9) |
| 16 |  |  | *Author* (10)* |
| 17 |  |  | R&D (8) |
| 18 |  |  | **ISI (7)** |
| 19 |  |  | **Academ* (8)** |
| 20 |  |  | Analy* (16) |
| 21 |  |  | Excell* (6) |
| 22 |  |  | **Produc* (12)** |
| 23 |  |  | **Scholar* (7)** |
| 24 |  |  | *Institut* (14)* |
| The * indicates a term where all variants after the * are possible, e.g. scien*: science, sciences, scientific, scientist, scientists, scientometrics, scientometrician, scientometricians.  The number of occurrences of all variants of a keyword is given between brackets. | | | |
| *Table 1: Search terms for Scientometrics 2003-2006* | | | |

In total, $360+30=390$ combinations of keywords were incorporated in the search string. This search string was used in the ThomsonReuters Web of Science online to find additional papers in the 42 issues of the 56 – 69 volumes of *Scientometrics*, published from January 2003 until December 2006. This yielded an additional 177 papers. Again, the titles and abstracts of the identified papers were considered and if the use of research indicators was mentioned, the paper itself was scrutinized and accepted as material for further use in the classification.

Results

It turned out that twelve papers in these additional volumes actually refer to the use of research indicators in addition to the 26 papers found manually. On average, this is less than the number of contributions found in the volumes searched manually (one paper per 2.88 issues for the manual searching method vs. one paper per 3.5 issues for the keywords based searching method). Of course, this difference might also reflect a slight growth in the attention to indicator use in *Scientometrics*. The combination of both search methods resulted in the identification of 38 contributions to *Scientometrics* during the years 2003 until 2013 which describe the use of research indicators. These 38 contributions represent 2.29% of the total amount of articles, letters and reviews published in *Scientometrics* in this period (n=1661). The selected articles describe a broad range of practices in many different countries mainly in Europe, but in Asia, South-America and Africa as well.

As described in the methods section of the paper, we did not only search contributions in *Scientometrics*. In order to create a comprehensive overview and to avoid limitations of *Scientometrics* we also paid attention to publications in other journal: *Higher Education, JASIST, Journal of Informetrics, Minerva, Research Evaluation*, *Research Policy*, *Science and Public Policy,* and *Social Studies of Science*. Moreover, we analyzed the *PLOS ONE* sections ‘Science policy’, ‘Research assessment’ and ‘Library science’. These sections partly overlap, but 176, 863, and 35 contributions were assigned to these sections respectively on the final date of our analysis of *PLOS ONE* (March 4^th^ 2014). In total, 299 issues of the listed journals except *PLOS ONE* were browsed (see table 2). We selected 59 papers which contained relevant information on the use of research indicators (one paper per 5.07 issues excluding *PLOS ONE*)

An overview of the results is given in table 2.

| Journal | Issues | First issue | Last issue | Total papers (articles, letters, reviews) | Relevant papers |  |
| --- | --- | --- | --- | --- | --- | --- |
| Higher Education | 60 | 57(1) | 66(6) | 456 | 15 | 3.29% |
| JASIST | 60 | 60(1) | 64(12) | 970 | 6 | 0.62% |
| Journal of Informetrics | 16 | 4(1) | 7(4) | 312 | 8 | 2.56% |
| Minerva | 22 | 47(1) | 52(2) | 121 | 4 | 3.31% |
| PLOS ONE |  |  |  |  | 0 | 0.00% |
| Research Evaluation | 29 | 16(1) | 22(1) | 202 | 11 | 5.45% |
| Research Policy | 50 | 38(1) | 42(10) | 626 | 11 | 1.76% |
| Science and Public Policy | 42 | 36(1) | 40(6) | 303 | 3 | 0.99% |
| Social Studies of Science | 20 | 40(1) | 43(2) | 124 | 0 | 0.00% |
|  |  |  |  |  |  |  |
| Total (excl. PLOS ONE) | 299 |  |  | 3102 | 59 | 1.92% |
|  |  |  |  |  |  |  |
| Scientometrics | 107 | 56(1) | 94(3) | 1661 | 38 | 2.29% |
|  |  |  |  |  |  |  |
| Total (incl. Scientometrics, excl. PLOS ONE) | 406 |  |  | 4763 | 97 | 2.04% |
| *Table 2: Overview of consulted sources* | | | | | | |

**3. Examples**

Only some examples of research indicator use are given in section 4 of the article. All other relevant material collected for this study is presented here. Forms of use can be assigned to more than one category. Next to examples from the publications as selected following the methods described in the section ‘methods’ we have used examples from the studies mentioned in the first section of this supplementary material (‘Overview of other contributions’) as a subsidiary source of examples.

A. General science policy

1. *General policy information:*

Delanghe et al. (2011) describe the development and use of bibliometric indicators by the European Commission for science policy at the European level (see also Lepori et al., 2008, p. 38). Indicators are also used at the European level for innovation policy (Zabala-Iturriagagoitia et al., 2007). Rankings can serve to inform about the status of the research system or university system (Geraci and Esposti, 2011: 668). In France, the *Observatoire de Science et des Techniques* (OST) produces indicators on topics such as international co-publications to create a general overview for policy-makers on internationalization, which enables France to detect developments in an early stage (Edler and Flanagan, 2011, p. 10; 13). Similarly, in the context of internationalization policies the Global Science and Innovation Forum (GSIF) in the UK includes bibliometrics in its reports on research performance of other countries (Edler and Flanagan, 2011, p. 11; 13). Another example is a ranking developed for the Taiwanese government to inform about the research performance of Taiwanese universities in comparison with other universities worldwide (Hou, 2011, p. 186).

1. *Policy formulation:*

The influence of indicators such as the Shanghai Jiao Tong University’s Academic Ranking of World Universities (ARWU) or the Times Higher Education Supplement THES-QS World University Rankings cannot be underestimated for policy making purposes. The French President Sarkozy ordered the French Minister of Science and Higher Education to set “the objective of having two French establishments in the top 20, and 10 in the top 100” (Anonymous, in: Hicks, 2012, p. 258). The minister gave herself until 2012 to achieve this. In order to improve the research capacity of French institutions, the French government determined to create centers of excellence. Strengthening research is also the aim of the German “Excellence Initiative”. Such policy initiatives seem to be inspired by world rankings (Saisana et al., 2011, p. 168). Rankings were also important for the Obama administration’s “R&D investments in top universities so that they remain globally pre-eminent” (Cantwell and Taylor, 2013, p. 201).

Rankings do not only play a role in policy-making in Western Europe and North America, but also in various Asian countries. In 2006, Taiwan used rankings to formulate its objective “to develop at least one university as one of the world’s top 100 universities in 5 years” (Hou et al., 2012, p. 27). Similar objectives were set for key departments and cross-university research centers (Hou, 2011, p. 186). Besides that, the Taiwanese government used bibliometric methods to distinguish research-oriented universities from other universities in order to develop policies aimed at making research intensive universities internationally excellent (Huang et al., 2006, p. 420). In the Chinese 985 Project, the People’s Republic of China’s government explicitly aims to have more publications in international journals by the participating universities (Zhang et al., 2013, p. 768). Part of the objectives of the Korean BK 21 policy have been formulated in terms of quantitative indicators: Korea should become a top 10 or top 9 producer of Science Citation Index (SCI) papers (Byun et al., 2013, p. 649; Cho and Palmer, 2013, p. 293).

1. *Policy evaluation:*

Bibliometric indicators were used in the evaluation of the Norwegian participation in the Framework Policies of the European Union (Langfeldt et al., 2012, p. 91). South Africa had a policy to support local journals financially. The responsible Department evaluated this policy in 1998 and compared two groups of journals: those which did receive financial support and those which did not. These two sets of journals were compared, amongst others, on the basis of their journal impact factors. The evaluation eventually led to the abolishment of journal funding (Pouris, 2005, pp. 214–6).

1. *Inducement:*

Two Dutch economists who operated under the nom de plume A. D. S. de Schuite initiated a ranking of economists and university departments of economics in The Netherlands. Their main purpose was to encourage Dutch researchers to publish in international top journals (in the field of economy) and to improve research performance of Dutch economists in general (Nederhof, 2008, pp. 164–165). Rankings in the United States by *US New & World Reports* or the National Academy are not linked to funding but do create prompts for higher performance (Hicks, 2012, p. 260). These rankings used bibliometric information in the appendices (Hicks, 2009, p. 394). Another example concerns the National Science Council of the Republic of China (Taiwan) which encourages the development of Taiwanese journals towards world-level journals by means of journal indicators. Kao et al. developed a journal ranking for the field of management for this purpose (Kao et al., 2008).

B. Funding allocation

1. *Formula-based block funding (FBBF)*

FBBF is applied in a number of countries. The best-known examples can be found in the UK, Australia, and New Zealand. The UK Research Assessment Exercise (RAE) takes place at the level of departments. The RAE scores have been used for FBBF (Rebora and Turri, 2013, pp. 1658–9; Harnad, 2009, pp. 152–3; Auranen and Nieminen, 2010, p. 826; Kenna and Berche, 2011, pp. 108–9; Hicks and Katz, 2011, p. 148; Ernø-Kjølhede and Hansson, 2011, p. 139; Barker, 2007, p. 4; Himanen et al., 2009, p. 427; Reidpath and Allotey, 2009, p. 786; Filippakou et al., 2010, pp. 547–548; Hicks, 2009, p. 396; Abramo et al., 2011, pp. 936–938; see also Williams, 1997). Australia has a long history with FBBF. Initially, it used the Composite Index with a strong focus on publication output (Hicks, 2009, p. 398). Its current Excellence for Research in Australia (ERA) is a partly based on research performance indicators such as the number of publications (Auranen and Nieminen, 2010, p. 826; Beerkens, 2013, p. 1680; Pontille and Torny, 2010, p. 349) and the ERA results are used to inform decisions on institutional block funding via the Sustainable Excellence Scheme (ARC, n.d.). In New Zealand’s Performance-Based Research Fund (PBRF) scheme, the funding formula is based on a quality score derived from peer review, external research funding and degree completions (Hodder and Hodder, 2010, pp. 887–8; Donovan and Butler, 2007, p. 234).

More recently, a number of continental European countries have implemented FBBF too. In Italy, a small share of institutional block funding is determined by indicators based on formulae. Two evaluation exercises have been conducted with this purpose: the Triennial Evaluation Exercise (VTR) and the Quality of Research Assessment (VQR). The share of funding allocated on the basis of indicators is, however, limited (Abramo et al., 2011; Abramo et al., 2011c, p. 231; Franceschet and Costantini, 2010, p. 542; Franceschet and Costantini, 2011, p. 277; Abramo et al., 2011a, p. 620; Abramo et al., 2012, p. 157; Reale et al., 2007). Moreover, changes in allocation are limited due to the incorporation of other indicators in the funding formula as well as limits on the effect the VTR could have on institutional funding (Rebora and Turri, 2013, pp. 1660–1). Universities in Flanders, Belgium, receive part of their funding on the basis of a formula: the BOF-key. A small part of block funding is determined by the research performance of institutions in terms of output and citation impact (Debackere and Glänzel, 2004). In the past, Flemish performance-based funding was based on Web of Science (WoS) data only, but since 2010 Flanders has implemented its own database for peer-reviewed non-WoS material such as books, book chapters, and proceedings (Ossenblok et al., 2012, p. 281; 288). The Danish Bibliometric Research Indicator assigns points for research output and quality; these points determine part of the block funding of research institutions (Ernø-Kjølhede and Hansson, 2011, p. 138). In the older Norwegian system a similar output and quality indicator is used for the level of block funding (Lepori et al., 2008, p. 38; Ossenblok et al., 2012, p. 281; Reale and Seeber, 2013, p. 140).

Norway and Denmark do not only use publication related indicators but also indicators measuring the level of external funding (Auranen and Nieminen, 2010, p. 827). External funding also is used or has been used in other European countries, such as Italy (Reale and Seeber, 2013, p. 140). In Germany, some states (*Länder*) allocate considerable parts of public funding via formulae in which the level of third-party funding is an important indicator.^[[2]](#footnote-2)^ Publication related indicators are at best of minor importance to most German universities (Schmoch and Schubert, 2009, p. 197). FBBF in Finland is based on two types of external funding but also on the number of centers of excellence and (Auranen and Nieminen, 2010, p. 827).

A non-European country with some FBBF is South Africa. South African institutions receive funding on the basis of the number of papers in governmentally approved journals or in Web of Science listed journals (Pouris, 2012, pp. 323–4; Matthews, 2013).

1. *Non-formula block funding:*

In our selection of papers, no examples of non-formula block funding could be identified. However, earlier contributions showed that Polish university departments are partly funded by the central government on the basis of the performance level at which they are put on the scale. Budgets are allocated on the basis of informed peer review (Hicks, 2012, p. 257).

1. *Additional funding, financial bonus or penalty*

Additional funding is often implemented by giving researchers individual bonuses, but institutions and even journals are also awarded bonuses. Probably the best known example is the Spanish *sexenio* system. The *sexenio* awards were created to stimulate the Spanish research system to catch up with the rest of the Western world: “The text of the Real Decreto itself (1086/1989) states that the ultimate objective of the new law is to foster university professors [sic!] research productivity and improve the diffusion of this research both nationally and internationally” (Jiménez-Contreras et al., 2003, p. 135). A *sexenio* is a type of bonus funding for individual researchers. Researchers can earn multiple *sexenios* during their career (Osuna et al., 2011, p. 578). Citations or the Journal Impact Factor are used in the deliberations for awarding a *sexenio* (Hicks, 2012, p. 254).

Payment for production has been introduced in a number of European countries at the institutional or individual level. Finnish university hospitals can earn a significant bonus for each article published in a high impact journal (Weingart, 2005, p. 118; 126). Russian Academy of Sciences researchers can receive a monthly financial bonus based on their research activity. Their research performance is measured by the number of publications and citations as well as the journal impact factor (Markusova et al., 2009, p. 250). Although Markusova et al. do not explain in detail how this system works, their text hints that different types of scientific contributions are given different scores. In Turkey, researchers are substantially rewarded by the Scientific and Technological Research Council of Turkey (TÜBİTAK) for individual publications. The reward depends on the Journal Impact Factor of the journal in which the paper is published (Önder and Kasapoğlu-Önder, 2011, p. 470).

Asian examples can be found in South Korea and in mainland China. South Korean universities can earn more governmental funding if they achieve quantitative objectives set by the government such as the number of articles published in the Science Citation Index (SCI) (Byun et al., 2013, p. 657). Sometimes, payment-for-performance is even more direct, such as individual cash bonuses in return for publications in countries such as South Korea or the PR of China (Van Dalen and Henkens, 2012, pp. 1282–3, see also: Byun et al., 2013, p. 657). Some Chinese universities let the amount of bonus funding depend on the impact factor or the type of journal such as *Nature* or *Science* (Shao and Shen, 2012, p. 201). Institutes of the Chinese Academy of Sciences can increase their innovation funds and their directors can obtain higher salaries if they perform well in a ranking created by the Academy of Sciences (Zhang et al., 2011, p. 878).

Mexican researchers can receive a temporary scholarships which increases their monthly income. Awarding of scholarships is based on peer review but depends on research productivity. The scholarship program distinguishes four grades and the level of the monthly additional funding depends on a the grade obtained (Galaz-Fontes and Gil-Antón, 2013, p. 361).

South Africa has a policy similar to the Spanish *sexenios:* researchers can be rated individually. The peer review based rating of a researcher determines the height of the additional financial support the researcher receives for research activities. Some South African universities increase the salary of rated researchers (Inglesi-Lotz and Pouris, 2011).

In our set of papers, we could not find examples of financial penalties. Still, from the earlier contributions it is clear that funding is related to contracts at the University of Twente (Jongbloed, 2011, p. 186). The university board could cut funding of institutes not reaching the agreed targets. This could also be seen as internal funding, which shows that the boundaries between the different categories are not watertight.

1. *Program and project funding*

Indicators can be used for many different types of program and project funding. Even if indicators are not explicitly required, indicators of esteem such as *sexenios* can be helpful in competitive funding applications (Hicks, 2012, p. 258; see also Jiménez-Contreras et al., 2003, p. 135). Explicit use of indicators for program and project funding are also reported in the literature. In 2008, bibliometric indicators and the results of peer reviews were used for the selection of Slovenian research project proposals. In this way, proposals which would be rejected anyway were separated from projects which would be peer reviewed in a second round (Južnič et al., 2010). The Turkish TÜBİTAK decides about project funding on the basis of the project proposal and the track record of the project leaders. Publications in indexed journals are an important indicator for evaluation of the past performance of project leaders (Önder and Kasapoğlu-Önder, 2011, p. 470). Funding decisions by the Australian Research Council are based on peer assessor ratings (Marsh et al., 2011, p. 171).

In Taiwan, journal indicators are used to inform the National Science Council of the Republic of China regarding the status of researchers who apply for grants (Kao et al., 2008, p. 97). This importance of journal publications is confirmed by another study, which states that “the National Science Council in Taipei (…) has routinely evaluated grant applications (…) according to the publication of SCI papers” (Lin and Chen, 2006, p. 514).

Brazilian researchers can apply for so-called “productivity fellowships”. The type of grant they can apply for is dependent on the fact if they already have earned a rating. Some types of grants are only open to researchers who were awarded a certain type of fellowship (Oliveira et al., 2012; Arruda et al., 2009). Moreover, in some Brazilian committees that judge research project proposals, the academic status of principal investigators was evaluated on the basis of research indicators (Zanotto, 2006, pp. 176–7). Argentinean researchers can apply for individual grants which can be used for financing facilities, instruments, resources, etc. Decisions with respect to such funding is based on ratings (Ubfal and Maffioli, 2011, p. 1272).

*9. Internal funding*

Data which has been collected and used at the national level is sometimes publicly available. Institutions can use such quantitative information for internal funding allocation (Hicks, 2012, p. 256). Internal funding models sometimes reflect national funding models (Beerkens, 2013, p. 1680). German universities use the share of third party funds to allocate funding internally. This measure is used by the *Länder* for funding allocation among universities too (Schmoch and Schubert, 2009, p. 197). Moreover, the number and level of grants obtained by German professors are used for negotiation of their merit-based salary (Musselin, 2013, p. 1170). In France, the grades obtained by laboratories and teaching programs in evaluations by AERES have been used for internal funding allocation by universities (Musselin, 2013, p. 1170). The University of Western Australia used a quantity-based funding formula which, in line with the national funding model, rewarded research output (Gläser et al., in: Weingart 2005, 126).

Sometimes, internal funding models are designed independently of national funding models. An example is Delft University of Technology which used journal impact factors for internal funding allocation (Abramo and D’Angelo, 2011, p. 349).

C. Organization and management

*10. Strategy*

Departments with low ratings in the UK RAE are sometimes reorganized or closed (Barker, 2007, p. 7). For the redefinition of the research funding priorities of the regional charitable trust (*Landesstiftung*) of Baden-Württemberg, bibliometric indicators were used (Grupp et al., 2009, pp. 550–1). In France, the grades laboratories and teaching programs obtained in evaluations by AERES have been used by universities to redesign research units (Musselin, 2013, p. 1170). Rankings such as the Shanghai ranking can help to formulate specific targets such as a position among the best 3 nationally and among the best 200 worldwide (Ylijoki, 2014, p. 71).

Outside of Europe, indicators play a role in strategic questions as well. For American universities, rankings are important tools for setting their strategic objectives (Cantwell and Taylor, 2013, p. 201). Taiwanese institutions are encouraged to use performance indicators from rankings for formulation of their institutional strategy formulation (Hou et al., 2012, pp. 40–41). It is not uncommon for universities to express their ambitions in terms of ranking positions. The University of KwaZulu-Natal has expressed the ambition to be “a top-ranked African university by 2016, as measured by recognized international higher education rankings” (University of KwaZulu-Natal in: Matthews, 2012, p. 677).

*11. Contract-based governance or steering*

In the set of papers we scrutinized for our classification we could not find examples on contract-based governance but earlier overviews do give such examples. Contract-based governance can take place at different levels. Jongbloed mentions the use of performance contracts for strategy formulation and quality management at Maastricht University (Jongbloed, 2011, pp. 183–188). Similar contracts existed in Denmark between the Ministry and the universities (Ab Iorwerth, 2005, p. 46).

*12. Accountability*

“Research performance data has been collected and monitored in [Australian] universities for almost two decades, ever since the government required universities to present data on publication numbers and on external grant funding” (Beerkens, 2013, p. 1680). The ARC admits that ERA results play a role in accountability to a wider public (ARC, n.d.). Moreover, Australian universities annually report data on their research output to the Department of Education, Science and Training (DEST) (Donovan and Butler, 2007, p. 233). In Taiwan, research indicators are used in the field of Chinese herbal medicine to make researchers accountable for their research performance in projects which are funded by the Department of Health (Lin and Chiang, 2007, pp. 70–72).

*13. Human resources management*

Vice-Chancelors in the UK indicate that managing human resources is very important for good RAE results (Goodall, 2009, pp. 1082–3). UK Business Schools use the journal ranking of the Association of Business Schools (ABS journal rankings) to inform staffing decisions for committees (Rafols et al., 2012, p. 1268). In the UK, job candidates are sometimes requested to provide bibliometric indicators next to their curriculum vitae (van Dalen and Henkens, 2012, p. 1283). Productive researchers are “headhunted” while retirement is made attractive for unproductive researchers (Rebora and Turri, 2013, p. 1659). A bad individual performance in the RAE can have consequences for career opportunities within an organization such as promotions or the amount of teaching (Leisyte et al., 2009, p. 626). In Italy, the *h-index* has become “the regulated reference threshold for access to a professorial career (…), both for candidates and for members of the national competition commissions” (Abramo et al., 2013, p. 199). In Spain, indicators at the individual level such as *sexenios* are used for tenure decisions (Hicks, 2012, p. 256; 258), and one or a number of *sexenios* are required for some professorships (Osuna et al., 2011) or for membership of commissions which grant tenure (Hicks, 2012, p. 258); see also (Jiménez-Contreras et al., 2003, p. 135). In this way, awarding *sexenios* distinguishes “active and competitive” researchers from researchers without a significant scientific contribution (Imperial and Rodríguez-Navarro, 2007, p. 272).

Research indicators do not only play a role in human resource management in Western European countries. The Turkish Interuniversity Council prescribes one or more publications in a WoS-based journal if a candidate wants to apply for an associate professorship. Besides that, institutes or departments have similar requirements next to the official governmental policy for both assistant professorships and full professorships (Gokceoglu et al., 2008, p. 420). Importance is attached to indexed journals when the research performance of candidates who apply for an assistant professorship is graded (Önder et al., 2008), although the importance of papers in Web of Science indexed journals differs from field to field (Önder and Kasapoğlu-Önder, 2011, p. 470).

In Taiwan too, promotion of faculty members is to a large extent based on publications in journals adopted by the Science Citation Index (SCI) (Lin and Chen, 2006, p. 514). If one applies for a professorship in mainland China, the minimum journal impact factor is used to distinguish papers which will be taken into account from contributions which will not be used in the assessment (Shao and Shen, 2012, pp. 200–1). In Brazil, researchers can be a member of CAPES and CNPq evaluation committees only if they have been awarded a certain type of research fellowship (Arruda et al., 2009, p. 653).

A remarkable example of research indicator use in human resources and careers was in the US. The supposedly discriminatory denial of tenure to a female professor was brought before a court and citation indicators served as an argument against the decision (Cronin in: Franceschet and Costantini, 2011, p. 283).

Thus, in a range of both traditional and upcoming research countries, research indicators are often used at the individual level to identify candidates who are eligible for a professorship or membership of a committee, to support the identification of the presumably best job candidate, or to decide on career opportunities of individuals within the organization.

*14. Quality management and quality assessment*

Sometimes, quality assessments are performed nationally but only concern a specific field, such as the field of Chemistry in the Netherlands. In 2001, the International Review Committee used bibliometric indicators to enable the quality assessment of chemistry research at Dutch universities by means of informed peer review (van Leeuwen et al., 2003, p. 258; Van Raan, 2005, p. 141). In general, the Dutch research assessment focuses on research quality for which indicators such as the number of honours, awards, prizes, and editorial and review activities are used (Donovan and Butler, 2007, p. 234). Finally, quality assessments are conducted at the individual level. In its evaluations, the French AERES uses journal ratings to assess the level and type of activity of academics in the laboratories it scrutinizes (Pontille and Torny, 2010, p. 352). Particularly institutions in the UK use bibliometric data in their evaluation reports (Bornmann, 2013, p. 727). For some French AERES journal lists, the journal impact factor has been used to determine the journal rating. These journal ratings should become tools for quality assessment (Pontille and Torny, 2010, pp. 355–6). Many Australian universities compare output information to assess their quality vis-à-vis other institutions in the country (Beerkens, 2013, p. 1680).

Quality assessment takes place at different levels. An example at the national level is the Australian ERA. The scientific production of Australian universities is evaluated and rated by committees. In each of the 180 Fields of Research a committee assesses the quality of their scientific activities. Research indicators such as bibliometric indicators and external research income are used for the evaluation of the research performance of institutes (Vanclay and Bornmann, 2012, p. 753; Franceschet and Costantini, 2011, p. 283; Abramo et al., 2011, p. 930). Thus, indicators are used for quality assessment itself in order to be able to give an appropriate rating.

An example at the institutional level is the Chinese Academy of Sciences, which developed a ranking of its institutes based on key performance indicators, such as the number of SCI papers, for “strengthening the regulation of S&T development”. Later, stricter evaluation criteria were applied, such as taking into account publications in highly cited SCI journals only (Zhang et al., 2011, p. 878). Another example of institutional level use of research indicators for quality assessment is the University of Pretoria. The University of Pretoria intends to measure its success in a number of academic activities by means of its position in the world rankings (Matthews, 2012, pp. 677–678).

Pillay generally states that indicators such as the *h-index* are sometimes used by heads of departments for individual level assessment (Pillay, 2013). Even if he does not exemplify his statement, an example of individual level application can be found in Thailand. The Thailand Research Fund assesses individual research quality by means of the so-called “Publication Credit”, which is the product of the Journal Impact Factor and the number of published research articles (Sombatsompop et al., 2005, p. 294; see also: Ventura and Mombrú, 2006, p. 288).

Indicators can also be used for a meta-purpose: the evaluation of evaluation procedures. In this way, metrics are used by the Vrije Universiteit Brussel to assess the reliability of peer-review evaluations (Rons et al., 2008, p. 56).

*15. Reputation management*

Even though national research assessments are mainly used for funding, their reputational importance cannot be underestimated. Institutions have indicated that they care more about the reputational effects of the ratings resulting from such such efforts than about the funding involved (Hicks, 2012, p. 258, see also: Rebora and Turri, 2013, p. 1659; Barker, 2007, p. 5; 10). The Spanish *sexenios* are a hallmark for scientific excellence, both for the individual researcher and for the research institute which hosts researchers with *sexenios* (Osuna et al., 2011); see also (Jiménez-Contreras et al., 2003, p. 135). In South Africa, the number of rated researchers is used to manage the reputation of universities (Inglesi-Lotz and Pouris, 2011).

Apart from awards, positions on rankings such as the Shanghai ranking can clearly play a role in the strategy of research institutions to improve their international status and visibility (Ylijoki, 2014, p. 71; Tofallis, 2012, p. 2). Many universities use such information to emphasize their national, regional, or global prominence. The following cases are only a few examples among many. Arizona State University “boasts of its position in global rankings” (Cantwell and Taylor, 2013, p. 201). South African universities mention their position in world rankings on their websites (Matthews, 2012, pp. 677–678). The Saudi Arabian King Saud University presents its worldwide and regional position in the QS World University Rankings, the Webometrics Rankings and the Shanghai Ranking (ARWU) on its website (Onsman, 2011, pp. 522–3).

Not only institutions but also publishers and journal editors use indicators for management of their reputation particularly by means of the Journal Impact Factor (Lozano et al., 2012, pp. 2140–1). Similarly, Australian journals such as *Australian Archaeology* publish their rating score as used in the ERA on its website (Pontille and Torny, 2010, p. 353).

*16. Selection of partners and members*

In the UK, industry and research funders sometimes use RAE ratings to find suitable partners (Barker, 2007, p. 7). The Association for Quality in Italian State Universities (AQUIS) aims amongst others to promote evaluation. Membership is only open to institutions which have at least a certain ranking position in the Shanghai Ranking (ARWU) and the THES World University Rankings (Rebora and Turri, 2011, p. 536).

D. Content management and decisions

*17. Publication channel selection*

Journal indicators may inform researchers on their choices for publication of their papers. Krauskopf points out that journal rankings are important for researchers to identify “prospecting” journals (Krauskopf, 2013). In general terms, Lozano et al. confirm such a role of the Journal Impact Factor for researchers (Lozano et al., 2012, pp. 2140–1). Moreover, a high journal impact factor is an important inducement for researchers not to publish in open access journals despite their support for the open access ideal (Volkmann et al., 2014, p. 207). Biomedical and clinical scientists in the study of Albert et al. have the impression that journals with a high impact factor apply stricter evaluation criteria. They believe that publishing in journals with a high status indicates originality of the contribution (Albert et al., 2012, p. 668).

A number of national initiatives serve researchers in publication related decisions. Australian researchers probably use the ERA journal ratings when they select the journals for publication of their contributions (e.g. Vanclay, 2011, p. 272, see also Pontille and Torny, 2010, p. 353). The function of the journal rankings created by the Association of Business Schools in the UK is only to inform authors about the best journals for publication (Rafols et al., 2012, p. 1268). Moreover, such indicators may even be imposed upon (junior) researchers. Chinese universities which participate in the 985 Project require their PhD students in science fields to publish a minimum number of papers in journals which are indexed in the Science Citation Index (SCI) or Engineering Index (EI) (Zhang et al., 2013, p. 773).

*18. Research profile management*

German universities have used DFG rankings to formulate their scientific priorities (Musselin, 2013, p. 1170). In the US, the National Research Council informs universities about their research profile by means of rankings of university departments (Abramo and D’Angelo, 2011, p. 348).

*19. Journal and database management*

The National Science Council of the Republic of China (Taiwan) uses research indicators to encourage the creation of world-level Taiwanese journals. For this purpose, Kao et al. (2008) developed a ranking of management journals. In the People’s Republic of China, grants for the development of scientific journals in China are awarded on the basis of citation, peer review, and publication related indicators (Shao and Shen, 2012, p. 201). Chinese databases use similar indicators for the adoption of journals in their indices (Shao and Shen, 2012, p. 202).

Of course, the Journal Impact Factor is an important concern of journal editors and this in turn steers the selection decisions of editors (Jarić, 2011). Moreover, Thomson Reuters, the publisher of products such as *Web of Science* and the *Journal Citation Reports,* uses research indicators to support its decision-making about the inclusion or exclusion of journals (Ferrara and Romero, 2013, p. 2333). Attempts at retrieval by researchers whose institutions do not have a right to access are used by publishers as download indicators in sales negotiations with those institutions (Volkmann et al., 2014, p. 195).

*20. Library collection management*

Indicators may play a role in content management decisions regarding library collections. Early citation studies had this purpose and this was also one of the original purposes of the Journal Impact Factor (Glänzel et al., 2006, p. 266). A recent example is the journal ranking by the UK-based Association of Business Schools which informs libraries on their purchases (Rafols et al., 2012, p. 1268). In mainland China, the Journal Impact Factor is used by librarians to guide them in their decisions on journal subscriptions (Shao and Shen, 2012, p. 202).

E. Consumer information

*21. Consumer information (not elsewhere specified)*

If results from national research assessments are made public, the media can use indicators to inform the general public about the national research performance (e.g. Hicks, 2012, p. 256). Moreover, students use such information to decide about their educational career, and researchers to choose promising places of employment (Hicks, 2012, p. 258; Saisana et al., 2011; Cantwell and Taylor, 2013, pp. 199–200; Zhu et al., 2004, p. 238; Tofallis, 2012, p. 2). Consequently, ranking positions have considerable effects on student behavior and institutional performance (Bowman and Bastedo, 2011, p. 432; 440).

Evaluation results by the UK RAE are made public and can be used by students and their families (Musselin, 2013, p. 1169; Barker, 2007, p. 5; Reidpath and Allotey, 2009, p. 796). Rankings partly based on research output (e.g. Sole 24 Ore) are included in university guides published by Italian media. This information is aimed at future students (Geraci and Degli Esposti, 2011, p. 668). Employers outside the research sector may use university rankings for recruitment decisions (Tofallis, 2012, p. 2). The Turkish ranking of universities created by the Higher Education Council of Turkey (YÖK) is based on article counts in indexed journals and attracts media attention. This ranking is important for students who pass the centralized university entrance examination and for researchers to find the best universities (Önder and Kasapoğlu-Önder, 2011, pp. 469–70).

**Bibliography**

Ab Iorwerth, A., 2005. Methods of Evaluating University Research Around the World (Working Papers-Department of Finance Canada No. 2005-04). Department of Finance Canada.

Abramo, G., Cicero, T., D’Angelo, C.A., 2012. The dispersion of research performance within and between universities as a potential indicator of the competitive intensity in higher education systems. Journal of Informetrics 6, 155–168. doi:10.1016/j.joi.2011.11.007

Abramo, G., Cicero, T., D’Angelo, C.A., 2011a. A field-standardized application of DEA to national-scale research assessment of universities. Journal of Informetrics 5, 618–628. doi:10.1016/j.joi.2011.06.001

Abramo, G., D’Angelo, C.A., 2011. National-scale research performance assessment at the individual level. Scientometrics 86, 347–364. doi:10.1007/s11192-010-0297-2

Abramo, G., D’Angelo, C.A., Costa, F.D., 2011b. National research assessment exercises: a comparison of peer review and bibliometrics rankings. Scientometrics 89, 929–941. doi:10.1007/s11192-011-0459-x

Abramo, G., D’Angelo, C.A., Costa, F.D., 2011c. National research assessment exercises: the effects of changing the rules of the game during the game. Scientometrics 88, 229–238. doi:10.1007/s11192-011-0373-2

Abramo, G., D’Angelo, C.A., Rosati, F., 2013. The importance of accounting for the number of co-authors and their order when assessing research performance at the individual level in the life sciences. Journal of Informetrics 7, 198–208. doi:10.1016/j.joi.2012.11.003

AERES, n.d. Evaluation campaigns [WWW Document]. Evaluation campaigns. URL http://www.aeres-evaluation.com/Evaluation/Discovering-the-evaluation-process/Evaluation-campaigns (accessed 4.12.14a).

AERES, n.d. Post-evaluation stages [WWW Document]. Post-evaluation stages. URL http://www.aeres-evaluation.com/Evaluation/Evaluation-of-research-units/Post-evaluation-stages (accessed 4.12.14b).

Albert, M., Laberge, S., McGuire, W., 2012. Criteria for assessing quality in academic research: the views of biomedical scientists, clinical scientists and social scientists. High Educ 64, 661–676. doi:10.1007/s10734-012-9519-2

ARC, n.d. Frequently Asked Questions [WWW Document]. URL http://www.arc.gov.au/era/faq.htm (accessed 2.26.15).

Arruda, D., Bezerra, F., Neris, V.A., Toro, P.R.D., Wainera, J., 2009. Brazilian computer science research: Gender and regional distributions. Scientometrics 79, 651–665. doi:10.1007/s11192-007-1944-0

Auranen, O., Nieminen, M., 2010. University research funding and publication performance—An international comparison. Research Policy 39, 822–834. doi:10.1016/j.respol.2010.03.003

Barker, K., 2007. The UK Research Assessment Exercise: the evolution of a national research evaluation system. Research Evaluation 16, 3–12. doi:10.3152/095820207X190674

Beerkens, M., 2013. Facts and fads in academic research management: The effect of management practices on research productivity in Australia. Research Policy 42, 1679–1693. doi:10.1016/j.respol.2013.07.014

Bornmann, L., 2013. The problem of citation impact assessments for recent publication years in institutional evaluations. Journal of Informetrics 7, 722–729. doi:10.1016/j.joi.2013.05.002

Bowman, N.A., Bastedo, M.N., 2011. Anchoring effects in world university rankings: exploring biases in reputation scores. High Educ 61, 431–444. doi:10.1007/s10734-010-9339-1

Box, S., 2010. Performance-based funding for public research in tertiary education institutions: Country experiences, in: Performance-Based Funding for Public Research in Tertiary Education Institutions: Workshop Proceedings. OECD Publishing, pp. 85–126.

Butler, L., 2010. Impacts of performance-based research funding systems: A review of the concerns and the evidence, in: Performance-Based Funding for Public Research in Tertiary Education Institutions: Workshop Proceedings. OECD Publishing, pp. 85–126.

Butler, L., 2004. What Happens when Funding Is Linked to Publication Counts?, in: Moed, H.F., Glänzel, W., Schmoch, U. (Eds.), Handbook of Quantitative Science and Technology Research. Kluwer Academic Publishers, Dordrecht, pp. 389–406.

Butler, L., 2003. Explaining Australia’s increased share of ISI publications—the effects of a funding formula based on publication counts. Research Policy 32, 143–155. doi:10.1016/S0048-7333(02)00007-0

Byun, K., Jon, J.-E., Kim, D., 2013. Quest for building world-class universities in South Korea: outcomes and consequences. High Educ 65, 645–659. doi:10.1007/s10734-012-9568-6

Cantwell, B., Taylor, B.J., 2013. Global Status, Intra-Institutional Stratification and Organizational Segmentation: A Time-Dynamic Tobit Analysis of ARWU Position Among U.S. Universities. Minerva 51, 195–223. doi:10.1007/s11024-013-9228-8

Cho, Y.H., Palmer, J.D., 2013. Stakeholders’ views of South Korea’s higher education internationalization policy. High Educ 65, 291–308. doi:10.1007/s10734-012-9544-1

Coryn, C.L.S., Hattie, J.A., Scriven, M., Hartmann, D.J., 2007. Models and Mechanisms for Evaluating Government-Funded Research An International Comparison. American Journal of Evaluation 28, 437–457. doi:10.1177/1098214007308290

Debackere, K., Glänzel, W., 2004. Using a bibliometric approach to support research policy making: The case of the Flemish BOF-key. Scientometrics 59, 253–276. doi:10.1023/B:SCIE.0000018532.70146.02

Delanghe, H., Sloan, B., Muldur, U., 2011. European research policy and bibliometric indicators, 1990–2005. Scientometrics 87, 389–398. doi:10.1007/s11192-010-0308-3

Donovan, C., Butler, L., 2007. Testing novel quantitative indicators of research “quality”, esteem and “user engagement”: an economics pilot study. Research Evaluation 16, 231–242. doi:10.3152/095820207X257030

Edler, J., Flanagan, K., 2011. Indicator needs for the internationalisation of science policies. Research Evaluation 20, 7–17. doi:10.3152/095820211X12941371876148

Ernø-Kjølhede, E., Hansson, F., 2011. Measuring research performance during a changing relationship between science and society. Research Evaluation 20, 131–143. doi:10.3152/095820211X12941371876544

Ferrara, E., Romero, A.E., 2013. Scientific impact evaluation and the effect of self-citations: Mitigating the bias by discounting the h-index. J Am Soc Inf Sci Tec 64, 2332–2339. doi:10.1002/asi.22976

Filippakou, O., Salter, B., Tapper, T., 2010. Compliance, resistance and seduction: reflections on 20 years of the funding council model of governance. High Educ 60, 543–557. doi:10.1007/s10734-010-9314-x

Franceschet, M., Costantini, A., 2011. The first Italian research assessment exercise: A bibliometric perspective. Journal of Informetrics 5, 275–291. doi:10.1016/j.joi.2010.12.002

Franceschet, M., Costantini, A., 2010. The effect of scholar collaboration on impact and quality of academic papers. Journal of Informetrics 4, 540–553. doi:10.1016/j.joi.2010.06.003

Galaz-Fontes, J.F., Gil-Antón, M., 2013. The impact of merit-pay systems on the work and attitudes of Mexican academics. High Educ 66, 357–374. doi:10.1007/s10734-013-9610-3

Geraci, M., Degli Esposti, M., 2011. Where do Italian universities stand? An in-depth statistical analysis of national and international rankings. Scientometrics 87, 667–681. doi:10.1007/s11192-011-0350-9

Geuna, A., Martin, B.R., 2003. University Research Evaluation and Funding: An International Comparison. Minerva 41, 277–304. doi:10.1023/B:MINE.0000005155.70870.bd

Glänzel, W., Debackere, K., Thijs, B., Schubert, A., 2006. A concise review on the role of author self-citations in information science, bibliometrics and science policy. Scientometrics 67, 263–277. doi:10.1007/s11192-006-0098-9

Gokceoglu, C., Okay, A.I., Sezer, E., 2008. International earth science literature from Turkey — 1970–2005: Trends and possible causes. Scientometrics 74, 409–423. doi:10.1007/s11192-007-1813-x

Goodall, A.H., 2009. Highly cited leaders and the performance of research universities. Research Policy 38, 1079–1092. doi:10.1016/j.respol.2009.04.002

Grupp, H., Hinze, S., Breitschopf, B., 2009. Defining regional research priorities: a new approach. Science and Public Policy 36, 549–559. doi:10.3152/030234209X469981

Hansen, H.F., 2010. Overview of models of performance-based research funding, in: Performance-Based Funding for Public Research in Tertiary Education Institutions: Workshop Proceedings. OECD Publishing, pp. 53–84.

Harnad, S., 2009. Open access scientometrics and the UK Research Assessment Exercise. Scientometrics 79, 147–156. doi:10.1007/s11192-009-0409-z

Hazelkorn, E., 2007. The Impact of League Tables and Ranking Systems on Higher Education Decision Making. Higher Education Management & Policy 19, 87–110.

Hicks, D., 2012. Performance-based university research funding systems. Research Policy 41, 251–261. doi:10.1016/j.respol.2011.09.007

Hicks, D., 2010. Overview of models of performance-based research funding, in: Performance-Based Funding for Public Research in Tertiary Education Institutions: Workshop Proceedings. OECD Publishing, pp. 23–52.

Hicks, D., 2009. Evolving regimes of multi-university research evaluation. High Educ 57, 393–404. doi:10.1007/s10734-008-9154-0

Hicks, D., Katz, J.S., 2011. Equity and Excellence in Research Funding. Minerva 49, 137–151. doi:10.1007/s11024-011-9170-6

Himanen, L., Auranen, O., Puuska, H.-M., Nieminen, M., 2009. Influence of research funding and science policy on university research performance: A comparison of five countries. Science and Public Policy 36, 419–430. doi:10.3152/030234209X461006

Hodder, A.P.W., Hodder, C., 2010. Research culture and New Zealand’s performance-based research fund: some insights from bibliographic compilations of research outputs. Scientometrics 84, 887–901. doi:10.1007/s11192-010-0201-0

Hou, A.Y.-C., 2011. Quality assurance at a distance: international accreditation in Taiwan. High Educ 61, 179–191. doi:10.1007/s10734-010-9331-9

Hou, A.Y.-C., Ince, M., Chiang, C.-L., 2012. A reassessment of Asian pacific excellence programs in higher education: the Taiwan experience. Scientometrics 92, 23–42. doi:10.1007/s11192-012-0727-4

Huang, M.-H., Chang, H.-W., Chen, D.-Z., 2006. Research evaluation of research-oriented universities in Taiwan from 1993 to 2003. Scientometrics 67, 419–435. doi:10.1556/Scient.67.2006.3.6

Imperial, J., Rodríguez-Navarro, A., 2007. Usefulness of Hirsch’s h-index to evaluate scientific research in Spain. Scientometrics 71, 271–282. doi:10.1007/s11192-007-1665-4

Inglesi-Lotz, R., Pouris, A., 2011. Scientometric impact assessment of a research policy instrument: the case of rating researchers on scientific outputs in South Africa. Scientometrics 88, 747–760. doi:10.1007/s11192-011-0440-8

Jarić, I., 2011. The use of h-index for the assessment of journals’ performance will lead to shifts in editorial policies. J. Am. Soc. Inf. Sci. 62, 2546–2546. doi:10.1002/asi.21642

Jiménez-Contreras, E., de Moya Anegón, F., López-Cózar, E.D., 2003. The evolution of research activity in Spain: The impact of the National Commission for the Evaluation of Research Activity (CNEAI). Research Policy 32, 123–142. doi:10.1016/S0048-7333(02)00008-2

Jongbloed, B., 2011. Funding through contracts, in: Enders, J., de Boer, H.F., Westerheijden, D.F. (Eds.), Reform of Higher Education in Europe. Sense Publishers, Rotterdam, pp. 173–191.

Jongbloed, B., Vossensteyn, H., 2001. Keeping up Performances: An international survey of performance-based funding in higher education. Journal of Higher Education Policy and Management 23, 127–145. doi:10.1080/13600800120088625

Južnič, P., Pečlin, S., Žaucer, M., Mandelj, T., Pušnik, M., Demšar, F., 2010. Scientometric indicators: peer-review, bibliometric methods and conflict of interests. Scientometrics 85, 429–441. doi:10.1007/s11192-010-0230-8

Kao, C., Lin, H.-W., Chung, S.-L., Tsai, W.-C., Chiou, J.-S., Chen, Y.-L., Chen, L.-H., Fang, S.-C., Pao, H.-L., 2008. Ranking Taiwanese management journals: A case study. Scientometrics 76, 95–115. doi:10.1007/s11192-007-1895-5

Kenna, R., Berche, B., 2011. Critical mass and the dependency of research quality on group size. Scientometrics 86, 527–540. doi:10.1007/s11192-010-0282-9

Kenna, R., Berche, B., 2011. Normalization of peer-evaluation measures of group research quality across academic disciplines. Research Evaluation 20, 107–116. doi:10.3152/095820211X12941371876625

Krauskopf, E., 2013. Deceiving the research community through manipulation of the impact factor. J Am Soc Inf Sci Tec 64, 2403–2403. doi:10.1002/asi.22905

Langfeldt, L., Godø, H., Gornitzka, Å., Kaloudis, A., 2012. Integration modes in EU research: Centrifugality versus coordination of national research policies. Science and Public Policy 39, 88–98. doi:10.1093/scipol/scs001

Laudel, G., 2005. Is external research funding a valid indicator for research performance? Research Evaluation 14, 27–34. doi:10.3152/147154405781776300

Leisyte, L., Enders, J., Boer, H. de, 2009. The balance between teaching and research in Dutch and English universities in the context of university governance reforms. High Educ 58, 619–635. doi:10.1007/s10734-009-9213-1

Lepori, B., Barré, R., Filliatreau, G., 2008. New perspectives and challenges for the design and production of S&T indicators. Research Evaluation 17, 33–44. doi:10.3152/095820208X291176

Liefner, I., 2003. Funding, resource allocation, and performance in higher education systems. Higher Education 46, 469–489. doi:10.1023/A:1027381906977

Lin, C., Chiang, C., 2007. Evaluating the performance of sponsored Chinese herbal medicine research. Scientometrics 70, 67–84. doi:10.1007/s11192-007-0105-9

Lin, M.-H., Chen, L.-K., 2006. The impact of impact factor on small specialties: A case study of family medicine in Taiwan. Scientometrics 66, 513–520. doi:10.1007/s11192-006-0037-9

Lozano, G.A., Larivière, V., Gingras, Y., 2012. The weakening relationship between the impact factor and papers’ citations in the digital age. J Am Soc Inf Sci Tec 63, 2140–2145. doi:10.1002/asi.22731

Markusova, V.A., Jansz, M., Libkind, A.N., Libkind, I., Varshavsky, A., 2009. Trends in Russian research output in post-Soviet era. Scientometrics 79, 249–260. doi:10.1007/s11192-009-0416-0

Marsh, H.W., Jayasinghe, U.W., Bond, N.W., 2011. Gender differences in peer reviews of grant applications: A substantive-methodological synergy in support of the null hypothesis model. Journal of Informetrics 5, 167–180. doi:10.1016/j.joi.2010.10.004

Matthews, A.P., 2013. Physics publication productivity in South African universities. Scientometrics 95, 69–86. doi:10.1007/s11192-012-0842-2

Matthews, A.P., 2012. South African universities in world rankings. Scientometrics 92, 675–695. doi:10.1007/s11192-011-0611-7

Molas-Gallart, J., 2012. Research Governance and the Role of Evaluation A Comparative Study. American Journal of Evaluation 33, 583–598. doi:10.1177/1098214012450938

Musselin, C., 2013. How peer review empowers the academic profession and university managers: Changes in relationships between the state, universities and the professoriate. Research Policy 42, 1165–1173. doi:10.1016/j.respol.2013.02.002

Nederhof, A.J., 2008. Policy impact of bibliometric rankings of research performance of departments and individuals in economics. Scientometrics 74, 163–174. doi:10.1007/s11192-008-0109-0

Oliveira, E.A., Colosimo, E.A., Martelli, D.R., Quirino, I.G., Oliveira, M.C.L., Lima, L.S., Silva, A.C.S. e, Martelli-Júnior, H., 2012. Comparison of Brazilian researchers in clinical medicine: are criteria for ranking well-adjusted? Scientometrics 90, 429–443. doi:10.1007/s11192-011-0492-9

Önder, Ç., Kasapoğlu-Önder, R., 2011. Resource endowments and responses to regulatory pressure: Publications of economics, management, and political science departments of Turkish universities in indexed journals, 2000–2008. High Educ 61, 463–481. doi:10.1007/s10734-010-9341-7

Önder, Ç., Sevkli, M., Altinok, T., Tavukçuoǧlu, C., 2008. Institutional change and scientific research: A preliminary bibliometric analysis of institutional influences on Turkey’s recent social science publications. Scientometrics 76, 543–560. doi:10.1007/s11192-007-1878-6

Onsman, A., 2011. It is better to light a candle than to ban the darkness: government led academic development in Saudi Arabian universities. High Educ 62, 519–532. doi:10.1007/s10734-010-9402-y

Orr, D., 2004. Research Assessment as an Instrument for Steering Higher Education — A Comparative Study. Journal of Higher Education Policy and Management 26, 345–362. doi:10.1080/1360080042000290195

Ossenblok, T.L.B., Engels, T.C.E., Sivertsen, G., 2012. The representation of the social sciences and humanities in the Web of Science--a comparison of publication patterns and incentive structures in Flanders and Norway (2005-9). Research Evaluation 21, 280–290. doi:10.1093/reseval/rvs019

Osuna, C., Cruz-Castro, L., Sanz-Menéndez, L., 2011. Overturning some assumptions about the effects of evaluation systems on publication performance. Scientometrics 86, 575–592. doi:10.1007/s11192-010-0312-7

Pillay, A., 2013. Academic promotion and the h-index. J Am Soc Inf Sci Tec 64, 2598–2599. doi:10.1002/asi.22998

Pontille, D., Torny, D., 2010. The controversial policies of journal ratings: evaluating social sciences and humanities. Research Evaluation 19, 347–360. doi:10.3152/095820210X12809191250889

Pouris, A., 2012. Scientometric research in South Africa and successful policy instruments. Scientometrics 91, 317–325. doi:10.1007/s11192-011-0581-9

Pouris, A., 2005. An assessment of the impact and visibility of South African journals. Scientometrics 62, 213–222. doi:10.1007/s11192-005-0015-7

Rafols, I., Leydesdorff, L., O’Hare, A., Nightingale, P., Stirling, A., 2012. How journal rankings can suppress interdisciplinary research: A comparison between Innovation Studies and Business & Management. Research Policy 41, 1262–1282. doi:10.1016/j.respol.2012.03.015

Reale, E., Barbara, A., Costantini, A., 2007. Peer review for the evaluation of academic research: lessons from the Italian experience. Research Evaluation 16, 216–228. doi:10.3152/095820207X227501

Reale, E., Seeber, M., 2013. Instruments as empirical evidence for the analysis of Higher Education policies. High Educ 65, 135–151. doi:10.1007/s10734-012-9585-5

Rebora, G., Turri, M., 2013. The UK and Italian research assessment exercises face to face. Research Policy 42, 1657–1666. doi:10.1016/j.respol.2013.06.009

Rebora, G., Turri, M., 2011. Critical factors in the use of evaluation in Italian universities. High Educ 61, 531–544. doi:10.1007/s10734-010-9347-1

Reidpath, D.D., Allotey, P., 2009. Can national research assessment exercises be used locally to inform research strategy development? The description of a methodological approach to the UK RAE 2008 results with a focus on one institution. High Educ 59, 785–797. doi:10.1007/s10734-009-9280-3

Rons, N., Bruyn, A.D., Cornelis, J., 2008. Research evaluation per discipline: a peer-review method and its outcomes. Research Evaluation 17, 45–57. doi:10.3152/095820208X240208

Saisana, M., d’ Hombres, B., Saltelli, A., 2011. Rickety numbers: Volatility of university rankings and policy implications. Research Policy, Special Section on Heterogeneity and University-Industry Relations 40, 165–177. doi:10.1016/j.respol.2010.09.003

Schmoch, U., Schubert, T., 2009. Sustainability of incentives for excellent research — The German case. Scientometrics 81, 195–218. doi:10.1007/s11192-009-2127-y

Shao, J., Shen, H., 2012. Research assessment and monetary rewards: the overemphasized impact factor in China. Research Evaluation 21, 199–203. doi:10.1093/reseval/rvs011

Simon, D., Knie, A., 2013. Can evaluation contribute to the organizational development of academic institutions? An international comparison. Evaluation 19, 402–418. doi:10.1177/1356389013505806

Sombatsompop, N., Markpin, T., Yochai, W., Saechiew, M., 2005. An evaluation of research performance for different subject categories using Impact Factor Point Average (IFPA) index: Thailand case study. Scientometrics 65, 293–305. doi:10.1007/s11192-005-0275-2

Tofallis, C., 2012. A different approach to university rankings. High Educ 63, 1–18. doi:10.1007/s10734-011-9417-z

Ubfal, D., Maffioli, A., 2011. The impact of funding on research collaboration: Evidence from a developing country. Research Policy 40, 1269–1279. doi:10.1016/j.respol.2011.05.023

Vanclay, J.K., 2011. An evaluation of the Australian Research Council’s journal ranking. Journal of Informetrics 5, 265–274. doi:10.1016/j.joi.2010.12.001

Vanclay, J.K., Bornmann, L., 2012. Metrics to evaluate research performance in academic institutions: a critique of ERA 2010 as applied in forestry and the indirect H2 index as a possible alternative. Scientometrics 91, 751–771. doi:10.1007/s11192-012-0618-8

Van Dalen, H.P., Henkens, K., 2012. Intended and unintended consequences of a publish-or-perish culture: A worldwide survey. Journal of the American Society for Information Science and Technology 63, 1282–1293. doi:10.1002/asi.22636

Van Leeuwen, T.N., Visser, M.S., Moed, H.F., Nederhof, T.J., Raan, A.F.J.V., 2003. The Holy Grail of science policy: Exploring and combining bibliometric tools in search of scientific excellence. Scientometrics 57, 257–280. doi:10.1023/A:1024141819302

Van Raan, A.F.J., 2005. Fatal attraction: Conceptual and methodological problems in the ranking of universities by bibliometric methods. Scientometrics 62, 133–143. doi:10.1007/s11192-005-0008-6

Ventura, O.N., Mombrú, A.W., 2006. Use of bibliometric information to assist research policy making. A comparison of publication and citation profiles of Full and Associate Professors at a School of Chemistry in Uruguay. Scientometrics 69, 287–313. doi:10.1007/s11192-006-0154-5

Volkmann, U., Schimank, U., Rost, M., 2014. Two Worlds of Academic Publishing: Chemistry and German Sociology in Comparison. Minerva 52, 187–212. doi:10.1007/s11024-014-9251-4

Weingart, P., 2005. Impact of bibliometrics upon the science system: Inadvertent consequences? Scientometrics 62, 117–131. doi:10.1007/s11192-005-0007-7

Williams, N., 1997. U.K. Universities—Jostling for Rank. Science 275, 18–19. doi:10.1126/science.275.5296.18

Ylijoki, O.-H., 2014. University Under Structural Reform: A Micro-Level Perspective. Minerva 52, 55–75. doi:10.1007/s11024-014-9246-1

Zabala-Iturriagagoitia, J.M., Jiménez-Sáez, F., Castro-Martínez, E., Gutiérrez-Gracia, A., 2007. What indicators do (or do not) tell us about Regional Innovation Systems. Scientometrics 70, 85–106. doi:10.1007/s11192-007-0106-8

Zanotto, E.D., 2006. The scientists pyramid. Scientometrics 69, 175–181. doi:10.1007/s11192-006-0134-9

Zhang, D., Banker, R.D., Li, X., Liu, W., 2011. Performance impact of research policy at the Chinese Academy of Sciences. Research Policy 40, 875–885. doi:10.1016/j.respol.2011.03.010

Zhang, H., Patton, D., Kenney, M., 2013. Building global-class universities: Assessing the impact of the 985 Project. Research Policy 42, 765–775. doi:10.1016/j.respol.2012.10.003

Zhu, X., Wu, Q., Zheng, Y., Ma, X., 2004. Highly cited research papers and the evaluation of a research university: A case study: Peking University 1974–2003. Scientometrics 60, 237–347. doi:10.1023/B:SCIE.0000027795.69665.09

1. For some countries, the share of performance based funding is very limited, but the reputational effects can be considerable or are deemed even more important (Hansen, 2010, pp. 40–2). [↑](#footnote-ref-1)
2. The logic behind third party funding is that it induces researchers to apply for external research grants (Weingart, 2005, p. 125). [↑](#footnote-ref-2)
